# A Highly Sensitive Time-Gated Fluorescence Immunoassay Platform Using Mn-Doped AgZnInS/ZnS Nanocrystals as Signal Transducers

**Published:** 2021-01-27

**Authors:** Brandon Gallian, Masoumeh Saber Zaeimian, Derrick Hau, David AuCoin, Xiaoshan Zhu

**Affiliations:** 1 Department of Electrical and Biomedical Engineering, University of Nevada, Reno, NV, United States,; 2 Biomedical Engineering Program, University of Nevada, Reno, NV, United States,; 3 Department of Microbiology and Immunology, School of Medicine, University of Nevada, Reno, NV, United States

**Keywords:** nanocrystals, Mn doping, time-gated fluorescence, immunoassay, analytical platform

## Abstract

In this work, a time-gated immunoassay platform using low-energy excitable and fluorescence long-lived Mn:AgZnInS/ZnS nanocrystals as signal transducers was developed and applied to the detection of the capsular polysaccharide (CPS) of *Burkholderia pseudomallei*, a Gram-negative bacterium that is the causative agent of melioidosis. CPS is a high molecular weight antigen displayed and is shed from the outer membrane of *B. pseudomallei*. The immunoassay using the time-gated platform presents a limit of detection at around 23 pg/ml when CPS is spiked in human serum.

## INTRODUCTION

Time-gated fluorescence measurement using long-lived fluorescence probes is a highly sensitive signal transduction method in biomedical research [1–5]. In the time-gated measurement, biological samples are excited using a pulsed optical source. After the excitation, autofluorescence from biological samples fades out quickly in tens to hundreds of nanoseconds. However, the long-lived probes continue emitting fluorescence up to milliseconds. Through detecting the time-gated emission from the probes, the signal-to-background ratio can be significantly enhanced to yield improved analytical sensitivity or a limit of detection (LOD).

The widely used probes for time-gated measurements mainly include lanthanide complexes or lanthanide-based nanoparticles, which have long fluorescence lifetimes at microseconds to milliseconds due to the unique energy bands (i.e., forbidden nature of the 4f transitions) of lanthanide elements [6]. For biosensing applications utilizing lanthanide-based probes, bulky laboratory-based instruments are routinely used for time-gated measurements; however, portable or small benchtop “personal” time-gated instruments are preferred to facilitate point-of-care or in-field testing [7–11]. One of the challenges is that the absorption spectra of lanthanide-based probes are generally in the ultraviolet (UV) range, and high-energy optical sources (e.g., 200–365 nm wavelength with tens or hundreds of milliwatts optical power) are needed to excite these probes in their applications. For instance, costly light-emitting diodes (LED) at 365 nm wavelength from Prizmatix (Mic-LEDs, > 200 mW, ~ $1,500) were reported as the excitation light sources for lanthanide-based probes [8]. Expensive UV-grade optics are also needed in order to avoid UV-induced (e.g., 365 nm) strong autofluorescence from glass/optics. Xenon lamps, broadly used in various instruments as excitation sources, are cheap but relatively large in size, and they need more strict operating conditions, including high current and kilovolts pulsed voltage to initiate light. Additionally, xenon lamps have a long trailing afterglow (hundreds of microseconds) but are hard to pulse rapidly [9, 10, 12]. UV laser diodes (e.g., <375 nm) as excitation sources are more expensive (~$4,000), and their output powers are also limited. As a result, the UV laser diodes may not be ideal excitation sources for lanthanide-based probes. High-energy optical sources not only complicate the instrument development but also increase the cost. It is important to find new high-quality probes for time-gated measurement under low-energy excitation.

Among various probes with long-lived luminescence for time-gated (or time-resolved) biosensing/imaging [13, 14], manganese- (Mn-) doped nanocrystals (NCs) including I(II)-III-VI NCs are attractive in biomedical research (i.e., biosensing/imaging) [15, 16]. Mn-doped I(II)-III-VI NCs can be synthesized in a relatively low temperature and a short time (<200°C, 1–2 h, an energy-saving procedure) and have low toxicity synthesis/handling/disposal. They also possess excellent optical properties, including high brightness and long fluorescence lifetimes resulting from the spin relaxation and slow inversion between ^4^T_1_ and ^6^A_1_ states of Mn. In spite of these merits, some recent works on Mn doping into I(II)-III-VI NCs show significant variations on their absorption/fluorescence spectra and lifetimes (hundreds of nanoseconds to milliseconds) of the doped NCs depending on the synthesis approaches/conditions [17, 18]. Recognizing that the electronic energy bands of the host NCs could interact with the energy levels of Mn dopants and thus determine the optical properties of Mn-doped quaternary NCs [15, 19], we have specifically investigated the interaction between Mn dopants and host AgZnInS/ZnS NCs by tuning host composition and also changing Mn spatial distribution and its concentration in host NCs [19]. Therefore, we achieved Mn-doped AgZnInS/ZnS (Mn:AgZnInS/ZnS) NCs with optimal optical properties, including long fluorescence lifetimes (1.33 milliseconds), low excitation energy (excitable at 405 nm wavelength), minimal emission reabsorption, and adequate brightness (>40% quantum yield). Compared to lanthanide-based probes, Mn:AgZnInS/ZnS NC-based probes would not replace them in all their applications. However, due to their unique optical properties and the availability of compact and cheap excitation sources at 405 nm wavelength with appropriate optical power outputs, Mn:AgZnInS/ZnS NC-based probes could be more efficient in facilitating the development of cost-effective small benchtop “personal” instruments. These instruments could have broader in-field sensing applications and avoid the need/use of central laboratories.

We have recently presented a compact time-gated fluorescence instrument using Mn:AgZnInS/ZnS NC-based probes as signal reporters and applied it for sensitive detection of copper(II) ions in highly autofluorescent rum (alcoholic beverage) [20]. Nevertheless, this system adopted a quartz cuvette with a volume of several milliliters as the sample holder and an optical path corresponding to such a cuvette. It is hard for this system to be used in microplate-based immunoassays, which can handle small sample volumes and also have wide applications in disease diagnosis, environmental monitoring, food safety analysis, and so on. It would be more interesting to develop a small and highly sensitive time-gated immunoassay platform using microplates and adopting Mn:AgZnInS/ZnS NC-based probes as signal transducers. Additionally, we have recently reported poly(maleic anhydride-alt-1-octadecene) (PMAO) based zwitterionic amphiphiles to encapsulate hydrophobic ligand coated nanoparticles for water solubility, minimal nonspecific binding, excellent colloidal stability, and bioconjugation [21]. In this work, we redesigned the instrument for the analysis/measurement on a small sample volume and also applied the zwitterionic amphiphiles to encapsulate hydrophobic Mn:AgZnInS/ZnS NCs to form water-soluble probes for immunoassay. We then mainly investigated whether such a redesigned instrument together with the Mn:AgZnInS ZnS NC-based probes can significantly enhance the immunoassay performance (i.e., lowering the LOD of immunoassay, which is a major advantage of time-gated measurement).

For the immunoassay, we utilized a high-affinity monoclonal antibody (mAb 4C4) to detect purified *B. pseudomallei* CPS spiked in human serum. mAb 4C4 was confirmed to be suitable for use in an antibody-based detection assay for the diagnosis of *B. pseudomallei* infections [22–24]. CPS is expressed on the cell envelopes of *B. pseudomallei*, which is listed as Tier 1 select agent by the CDC and is a Gram-negative bacterial pathogen affecting both human beings and animals to cause melioidosis [25, 26]. The immunoassay for the detection of CPS in human biological fluid can be potentially used in mobile laboratories for in-field melioidosis diagnosis. It should be noted that culture of *B. pseudomallei* from blood or other body fluids is still the gold standard for diagnosis of melioidosis, but it needs central laboratories and also takes 3–5 days before a definitive diagnosis can be made. Thus, developing a compact highly sensitive immunoassay platform for CPS detection is of important significance for melioidosis diagnosis and rapid administration of effective antibiotics.

## MATERIALS AND METHODS

The detailed experimental approaches/methods are described in the Supplementary Material.

## RESULTS AND DISCUSSION

**FIGURE 1 |:**
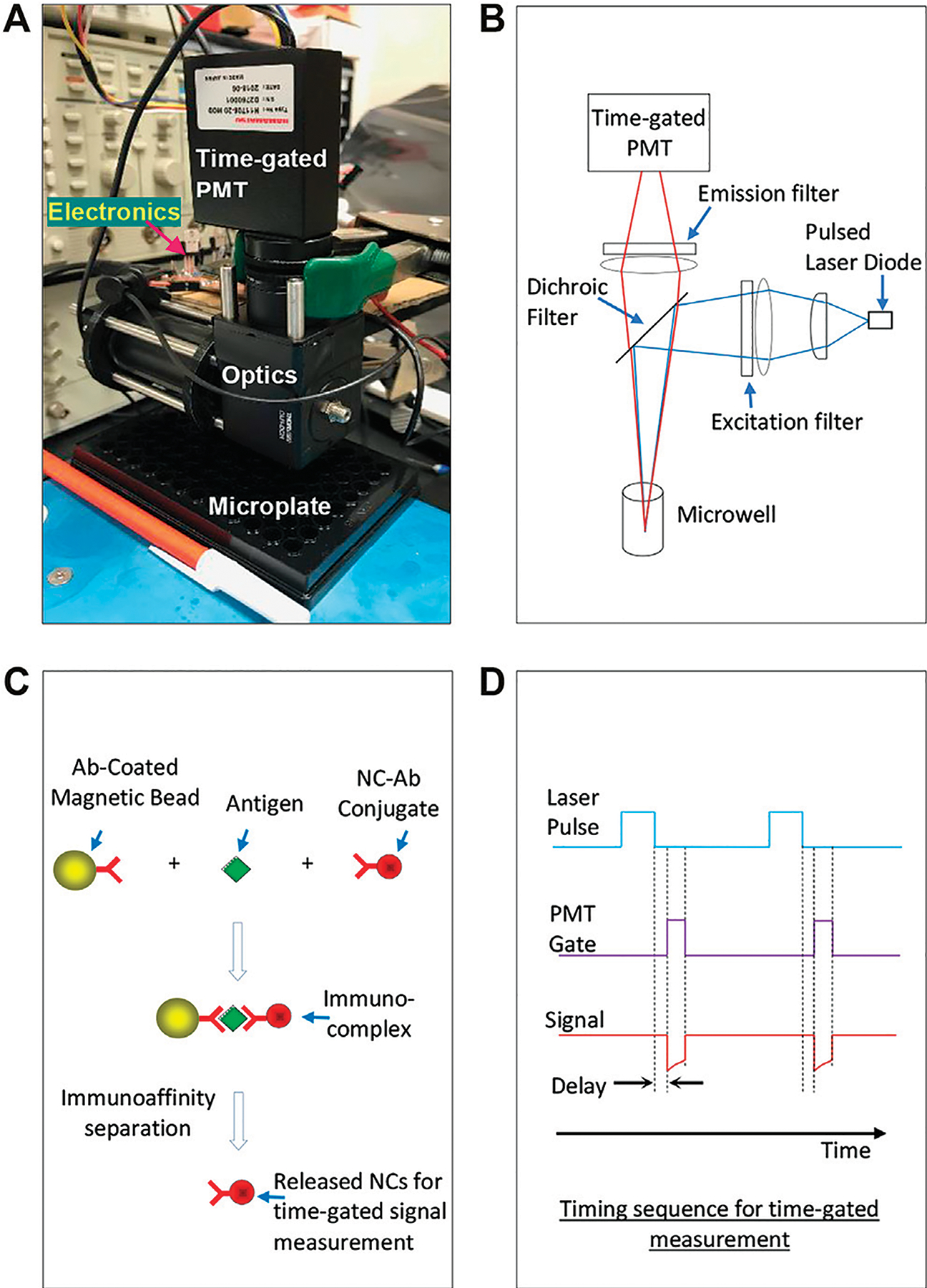
**(A)** A small time-gated immunoassay platform integrating a time-gated optical detector and microplate-based immunoassay using Mn:AgZnInS/ZnS NC probes as signal transducers. **(B)** Principle illustration of the optical unit. **(C)** The immunoassay format performed in microwells of microplates: after antigens are switched between antibody-coated magnetic microbeads and antibody-conjugated NC probes, the NC probes are released from the immuno-switch-complexes into supernatants for time-gated signal measurement. **(D)** The timing sequence for time-gated measurement on the excitation laser and the PMT.

Figure 1A presents the small time-gated immunoassay platform integrating a time-gated optical detector and a microplate-based immunoassay using Mn:AgZnInS/ZnS NC probes as signal transducers. Compared to our previously reported instrument [20], we kept all electronics but redesigned the optical path for microwell-based detection. Figure 1B illustrates the design of the optical unit using a dichroic-filter ZT514rc (Chroma) to split excitation and emission light. The dichroic filter reflects light with short wavelengths (<514 nm) to the sensing surface/volume but allows light with long wavelengths (>514 nm) to pass through the dichroic filter. On the excitation (or laser diode) side, one planoconvex lens and one biconvex lens direct the laser light to the dichroic filter. To avoid any possible autofluorescence from lenses and harmonic wavelengths from the laser diode, a bandpass optical filter (405 ± 10 nm, Chroma) filter is placed at the innermost end of the excitation-side slotted lens tube. On the microwell side, the reflected laser light is focused on a region of the microwell. The emission light from the sample passes through the dichroic filter, a biconvex lens, and an emission filter (655 ± 20 nm, Chroma) and then is focused into a time-gated photomultiplier tube (PMT). The whole optical path is built in two lens tubes and a dichroic-filter cage cube with necessary adapters. The PMT is directly placed at the end of a lens tube using a PMT-to-C-mount adapter, C-mount-to-SM1 adapter, and a silicone gasket to block out stray light at the junction of the PMT and C-mount adapter. An adapter surrounded with a rubber gasket (as a part of the optical detector) is used to align/seal with each microwell without touching samples loaded in the microwell. The microplate under the testing can be manually shifted; thus, each microwell can be read by the optical detector (with good alignment and sealing with the optical detector). Before the use of the instrument, with the continuous turn-on of the laser diode, the lenses in the excitation branch were manually adjusted/tuned in their positions to achieve the highest signal/background ratio for a probe solution with a certain concentration (e.g., 1 μg/ml) and a blank, which were loaded in a microwell with a certain volume (e.g., 100 μL). The cost for the optical detector, including optics and electronics, is ~$4,000, with the major spending on PMT and optical filters.

Figure 1C shows the immunoassay format performed in microwells as we reported before (Ref. 27). In principle, antigens are first switched between antibody-coated magnetic microbeads and antibody-conjugated NC probes, and then the probes are released from the switched immunocomplexes into supernatant using an appropriate immunoaffinity separation solution to denature antibodies in complexes. The released probes were separated from magnetic microbeads and suspended in the immunoaffinity separation solution for signal reading under the time-gated mode. The separation of NC probes from immunocomplexes can avoid the interference of magnetic beads in fluorescence measurement. In this immunoassay format, the suspended microbeads have advantages in efficiently capturing and separating antigens from complex sample matrices and facilitating all wash procedures in the assay. Figure 1D illustrates the timing sequence for time-gated measurement on the excitation laser and the PMT.

The Mn:AgZnInS/ZnS NCs were synthesized according to our previous report [19]. In the first step, Mn:AgZnInS were synthesized by using 0.2 mmol Zn precursor, 0.2 mmol In precursor, 0.025 mmol Mn precursor, 0.8 mmol sulfur precursor, and 0.05 mmol Ag precursor. After Mn atoms were homogenously doped into AgZnInS NCs, a ZnS shell was grown on NCs to form Mn:AgZnInS/ZnS NCs. Supplementary Figure S1 shows the material characterization of the prepared Mn:AgZnInS/ZnS NCs, including their energy-dispersive X-ray (EDX) spectrum and transmission electron microscopy (TEM) image. Supplementary Figure S2A presents the absorption and fluorescence spectra when these NCs were suspended in hexane. Supplementary Figure S2B shows the fluorescence decay of these NCs. It can be seen that >40% light intensity remains within the first 200 μs delay, which is good for time-gated measurement.

**FIGURE 2 |:**
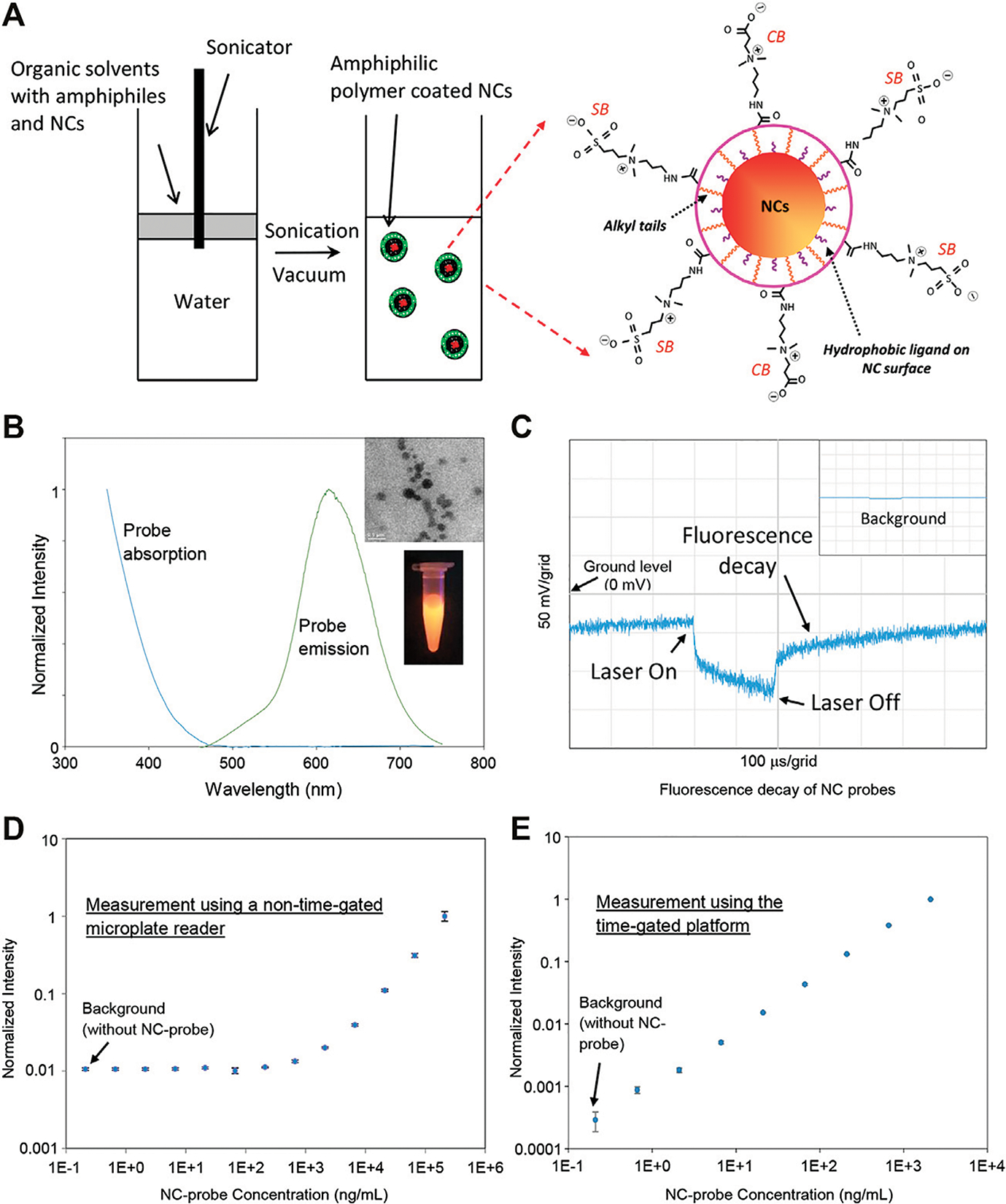
**(A)** Illustration on the preparation method of NC probes for immunoassay. **(B)** The fluorescence and absorption spectra of the NC probes. The inset images present a representative TEM image of the prepared NC probes and their emission under UV light. **(C)** Representative fluorescence decay of the NC probes diluted in pure water—the NC probes emit fluorescence after laser-off. The inset presents the background for pure water. **(D)** Calibration curve of the NC probes in water (with 5% BSA mimicking a high autofluorescent sample matrix) measured using a nongated microplate reader. **(E)** Calibration curve of the NC probes in water (with 5% BSA) measured using the time-gated platform.

These NCs were produced in organic solvents with naturally hydrophobic surfaces and needed to transfer water for further bioapplications. Poly(maleic anhydride-alt-1-octadecene) (PMAO) can be modified to incorporate zwitterions such as carboxybetaine (CB) and sulfobetaine (SB) on PMAO backbones to form amphiphilic polymer PMAO-CB-SB [21]. Here, we applied PMAO-CB-SB to Mn:AgZnInS/ZnS NCs for NC surface modification. Figure 2A illustrates the basic method for surface modification. PMAO-CB-SB and NCs mixed in an organic solvent were added on top of water. With sonication, organic droplets containing polymers and NCs were further broken down and dispersed into water. Followed up by rotary evaporation, organic solvents in the droplets were removed and self-assembly between PMAO-CB-SB and NCs occurred through hydrophobic interaction, while CB and SB groups are exposed to aqueous solutions. CB and SB groups coated on NC probes can prevent their aggregation and significantly lower their nonspecific binding because these zwitterions can interact with water molecules to form a stable hydration layer. CB can be further used for bioconjugation through covalent cross-linking chemistry using 1-ethyl-3-(3-dimethylaminopropyl)carbodiimide (EDC) and N-hydroxysulfosuccinimide (Sulfo-NHS) [27]. In the preparation of the NC probes, they were filtrated through a 0.2 μm membrane filter. Figure 2B presents the absorption and fluorescence spectra of the water-soluble NC probes and their TEM image and a digital image of these probes exposed under UV light. Figure 2C shows a representative decay of the diluted NC probes under a single pulse of a laser diode (1 kHz repetition with a 200 μs pulse width). As shown in this figure, the NC probes present a long-time fluorescence decay in the time domain after laser-off and the fluorescence of NC probes after laser-off does not totally fade out before the next moment of laser-on. The NC probes are suitable for the time-gated measurement (picking up a signal after the excitation light is off).

In order to demonstrate the detection capability of the time-gated platform, we compared the LODs between this platform and a standard non-time-gated microplate reader by measuring a series of diluted solutions with a probe concentration range from 0.66 ng/ml to 2.1 μg/ml in 5% bovine serum albumin (BSA). The use of 5% BSA is to mimic a high autofluorescent sample matrix. In this work, the non-time-gated microplate reader from PerkinElmer was used as a standard instrument, which is usually used in a central analytical laboratory. As shown in Figure 2D, the calibration curve established by the non-time-gated microplate reader (with 405 nm excitation) indicates a LOD at ~273 ng/ml. For the time-gated system, its electronics design/setting and the data processing of this platform are the same as what we reported [20]: the gain control voltage of PMT is at 1.080 V (a maximum value) to achieve the highest gain for the input light to current conversion, the laser diode is pulsed with 200 μs for excitation with a repetition rate of 1.024 kHz, and the delay time after laser-off is 50 μs, and the time period to pick up the signal (the detection window) is 100 μs. The calibration curve measured using this platform (with a 405 nm laser as excitation) is shown in Figure 2E and presents a LOD of ~354 pg/ml. Although two instruments have different optics and electronics, the difference in LODs clearly indicates that the time-gated platform as a standalone instrument is more sensitive or capable of detecting a lower level of NC probes because it can avoid the high autofluorescence from BSA.

The immunoassay for the detection of CPS in a microplate was developed in a format illustrated in Figure 1C. In the assay, anti-CPS mAb 4C4 was conjugated on both magnetic microbeads and the NC probes for CPS capture and detection (CPS has multiple binding sites), respectively. In this assay development, the experimental approach went through two steps. In the first step, using the non-time-gated microplate reader as the signal measurement tool, the assay was optimized and its calibration curve was then established. In the second step, the same microplates (used to set up the calibration curve under the measurement of the nongated microplate reader) was read out by our time-gated optical detector, and then the LODs by two different measurement tools were extracted and compared. Both instruments use the light at 405 nm wavelength for the probe excitation.

**FIGURE 3 |:**
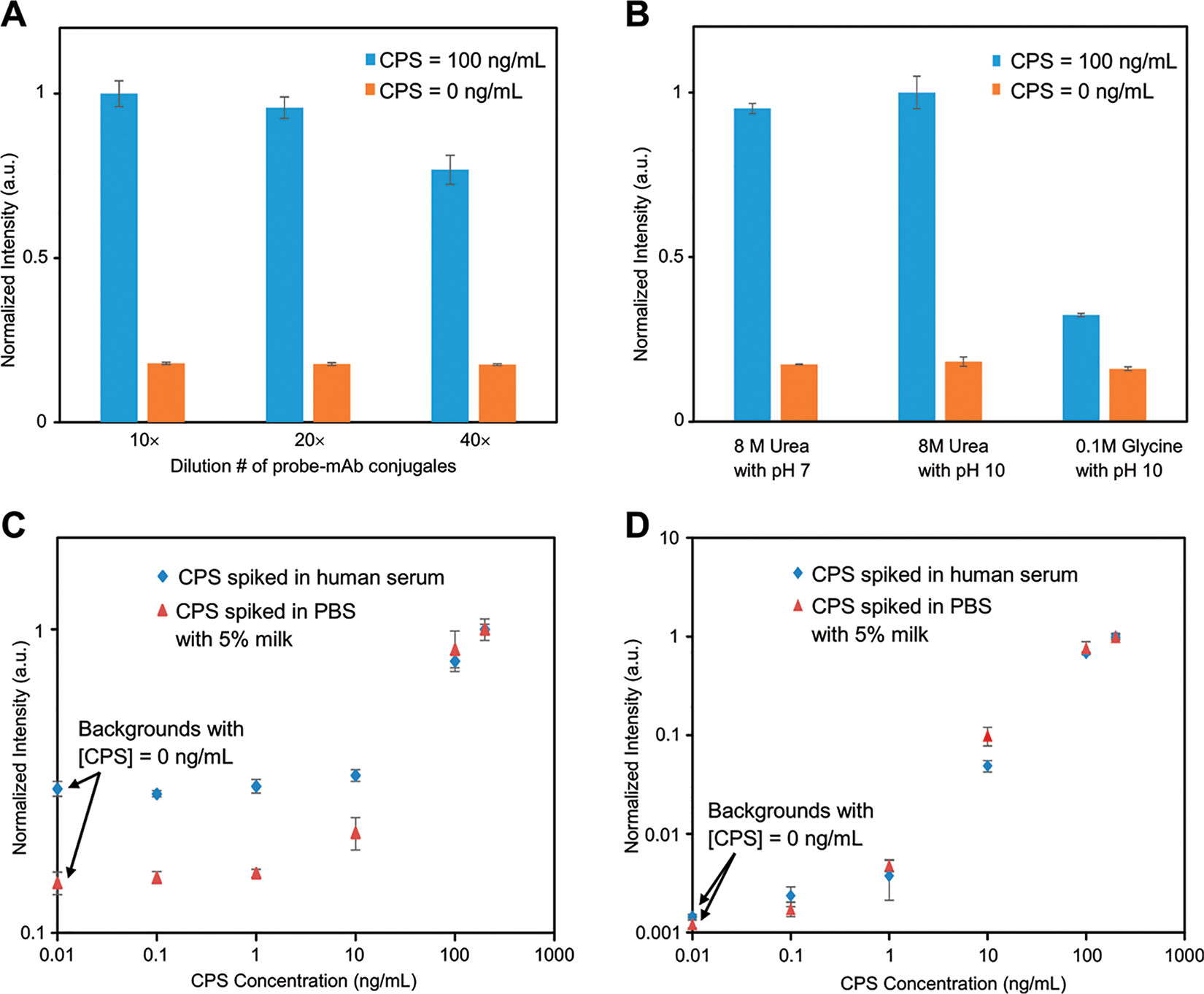
**(A)** Dilution optimization of antibody-coated NC probes for immunoassay. **(B)** Optimal selection of immunoaffinity separation solutions for immunoassay (all separation solutions contain 0.1% BSA and 1% SDS, and the optimization experiments were done with measurements using a standard non-time-gated microplate reader). **(C)** Calibration curves of immunoassays measured using a standard nongated microplate reader. **(D)** Calibration curves of immunoassays measured using the time-gated platform.

For assay optimization, two sets of magnetic microbeads were incubated with 0 ng/ml and 100 ng/ml CPS spiked in phosphate-buffered saline (PBS) with 5% milk, respectively. Afterward, conjugates (anti-CPS coated probes) diluted from the stock were applied to each microbead set to form immunocomplexes. The probes were released from the immunocomplexes for signal reading using an appropriate immunoaffinity separation solution to denature antibodies in the immunocomplexes. The readings from 0 ng/ml and 100 ng/ml CPS were designated as the background and the signal, respectively. In the assay optimization experiments, we mainly aimed to identify the dilution number of the stock conjugates and the appropriate immunoaffinity separation solution that can achieve higher signal/background ratios. Figure 3A presents the signals and the backgrounds when different dilutions of the stock conjugates were applied for assay labeling. It can be seen that 10× dilution of the stock conjugates can achieve a higher signal/background ratio. Thus, this dilution condition was used in the sequential assays. In the assay, an appropriate immunoaffinity separation solution is used to denature antibodies in switched immunocomplexes so that the NC probes can be released to separate from magnetic microbeads for signal measurement. The immunoaffinity separation solution is expected to efficiently release NC probes from magnetic beads and also stabilize the fluorescence signal of the released NC probes for reliable signal measurement. Several immunoaffinity separation solutions (8M urea with pH7, 8M urea with pH10, and 0.1M glycine with pH10) were tested. Note that these solutions are supplemented with 0.1% BSA and 1% sodium dodecyl sulfate (SDS) [27]. The fluorescence stability of the anti-CPS-conjugated NC probes was tested for 2 h with different dilutions of the stock conjugates in these separation solutions and are presented in Supplementary Figure S3, which indicates that all separation solutions present a good capability to stabilize the NC probes. Figure 3B shows the signals and the backgrounds when these immunoaffinity separation solutions were applied to release probes for signal reading. As shown in Figure 3B, the signal/background ratios using 8M urea (pH7) and 8M urea (pH10) are similar to each other without significant difference but higher than that using 0.1M glycine (pH 10). Although both 8M urea (pH7) and 8M urea (pH10) can be used, 8M urea (pH7) is easier to prepare, handle, and dispose due to its neutral pH and was used further.

With the optimized conditions, different concentrations of CPS spiked in PBS with 5% milk or human serum were prepared and assayed. Figure 3C presents two assay calibration curves when CPS was spiked in PBS with 5% milk and human serum, respectively. These calibration curves were established using the non-time-gated microplate reader as the measurement tool. From these curves, using the 3σ method, the calculated LODs of assays are ~5 ng/ml for CPS spiked in PBS with 5% milk and ~14 ng/ml for CPS in human serum. With triplicate running of the calibration experiments using the non-time-gated microplate reader, the LOD for CPS spiked in PBS with 5% milk presents an average value with a standard deviation at 9 ± 4 ng/ml, and the LOD for CPS spiked in human serum is at 10 ± 5 ng/ml. The same microplates used to set up the calibration curves in Figure 3C were also read out using the time-gated optical detector, and the calibration curves under the time-gated measurement are presented in Figure 3D. Through the direct comparison between the curves in Figures 3C,D, it can be clearly seen that the CPS concentrations at 0.1 ng/ml and 1 ng/ml are buried in the background under non-time-gated measurement but can be read out under time-gated measurement. From Figure 3D, the LODs of assays for CPS spiked in PBS with 5% milk and human serum are calculated to be ~65 pg/ml and ~23 pg/ml, respectively. With triplicate running of the calibration experiments using the time-gated instrument, the LOD for CPS spiked in PBS with 5% milk present an average value with a standard deviation at 64 ± 4 pg/ml, and the LOD for CPS spiked in human serum is at 23 ± 1 pg/ml. When compared to the non-time-gated microplate reader, the time-gated instrument as a standalone measurement tool can achieve a much lower LOD (at least two ordera lower). Through the assay development, it can be seen that the time-gated immunoassay for the highly sensitive detection of CPS in human biological fluid is feasible and has the potential to be used for melioidosis diagnosis.

After the time-gated assay calibration curve (with CPS spiked in human serum) was established, the assay reliability was tested by measuring the signals of 100 ng/ml CPS in human serum and then comparing them to the signal of 100 ng/ml CPS resulting from the calibration curve. The average recovery is ~91%, with a standard deviation at ~8.8%. The recoveries for triplicates of lower concentrations (1 ng/ml, 0.1 ng/ml) of CPS in human serum were also tested. The average recovery for 1 ng/ml CPS is ~94% with a standard deviation at ~9.9%, and the average recovery for 0.1 ng/ml CPS is ~89% with a standard deviation at ~0.5%. The assay accuracy reflected by the average recoveries is in the range of 89–94%. This study suggests that the time-gated immunoassay on the detection of CPS in human serum is reliable.

Supplementary Table S1 presents a comparison with several recently reported assays on CPS detection. An optimized lateral flow immunoassay (LFIA) using gold nanoparticles as probes/labels shows LOD at ~200 pg/ml [23, 28]. An enzyme-based microplate immunoassay using horseradish peroxidase (HRP) to react with substrates (3,3’,5,5’-tetramethylbenzidine (TMB) and H_2_O_2_) presents LOD at ~200 pg/ml [23]. A very recent LFIA using gold nanoparticles as labels presents LOD at ~20 pg/ml, but it was only tested using CPS spiked in PBS buffer with a large volume (~50 ml) [29]: 50 ml of CPS-spiked PBS buffer was filtrated and preconcentrated through a membrane. Please note that although the filtration/preconcentration approach is a very good way to enhance assay sensitivities, filtrating a 50 ml of human serum sample (or samples with complex matrices) could cause the membrane to be clogged very quickly. Our assay under the time-gated measurement used 50 μL of samples and can achieve a LOD at ~23 pg/ml.

## CONCLUSION

In this work, a time-gated immunoassay platform was developed and applied for the detection of CPS (a biomarker of melioidosis). This platform integrates a time-gated optical detector and microplate-based immunoassay using Mn:AgZnInS/ZnS NC probes as signal transducers. Compared to the immunoassay under non-time-gated measurement, the immunoassay measured by the time-gated optical detector presented more than two orders lower LOD at ~23 pg/ml (with CPS spiked in human serum) and broader detection range.

## Supplementary Material

Supplementary
