# Supplementary material for: A Highly Sensitive Time-Gated Fluorescence Immunoassay Platform Using Mn-Doped AgZnInS/ZnS Nanocrystals as Signal Transducers
Source: Front Phys. Author manuscript; Available in PMC 2021 Jul 1. (PMC8009851)
Supplement: Supplementary [file NIHMS1685094-supplement-Supplementary.pdf]

Supplementary

A Highly Sensitive Time-Gated Fluorescence Immunoassay Platform Using Mn Doped AgZnInS/ZnS Nanocrystals as Signal Transducers

Brandon Gallian, [a,c] Masoumeh Saber Zaeimian, [a,c] Derrick Hau, [b]

David AuCoin, [b] and Xiaoshan Zhu* [a,c]

[a] *Department of Electrical and Biomedical Engineering, University of Nevada, Reno, NV, USA*

[b] *Department of Microbiology and Immunology, University of Nevada, Reno, School of Medicine, NV, USA*

[c] *Biomedical Engineering Program, University of Nevada, Reno, NV, USA*

*To whom correspondence should be addressed. Email: xzhu@unr.edu. Fax: 1-775-784-6627

Materials and Methods

Materials

Bovine serum albumin (BSA) Fraction V was from MP Biomedicals. Urea, $NaH_2PO_4 \bullet H_2O$, $Na_2HPO_4 \bullet 2H_2O$, NaOH, HCl, $(NH_4)_2SO_4$, NaN_3, glycine, sodium dodecyl sulfate (SDS), and Tween 20 were from Fisher Scientific. 1-Ethyl-3-(3-dimethylaminopropyl)carbodiimide (EDC), and N-hydroxysulfosuccinimide (Sulfo-NHS) were purchased from Sigma Aldrich. Milk powder was purchased from Walmart. *Burkholderia pseudomallei* capsular polysaccharide (CPS) antigen and monoclonal anti-CPS antibodies were obtained from the School of Medicine at the University of Nevada Reno.

Buffer-1 is 0.1M Na-Phosphate buffer with pH 7.4. It was made by dissolving 0.262 g $NaH_2PO_4 \bullet H_2O$ and 1.442 g $Na_2HPO_4 \bullet 2H_2O$ into 100 mL DI water. Its pH was adjusted to 7.4 using NaOH pellets or 1N HCl solution. Buffer-2 is 3 M $(NH_4)_2SO_4$ in 0.1M Na-Phosphate buffer or Buffer-1. Simply, 3.964 g $(NH_4)_2SO_4$ was dissolved into 10 mL of 0.1M Na-Phosphate buffer or Buffer-1. Buffer-3 is PBS pH7.4 with 0.5% BSA. It was prepared by adding 0.88 g NaCl, 0.5 g BSA and 10 mL of 0.1M Na-Phosphate buffer or Buffer-1 into 90 mL DI water Buffer-4 is PBS pH7.4 with 0.1% BSA and 0.02% NaN_3. It was prepared through adding 0.88 g NaCl, 0.1 g BSA, 0.02 g NaN_3, and 10 mL of 0.1M Na-Phosphate buffer or Buffer-1 into 90 mL of DI water. Several immunoaffinity separation solutions including 8M urea with pH7, 8M urea with pH10, and 0.1M glycine with pH10 were prepared. All immunoaffinisty separation solutions were supplemented with 0.1% BSA and 1% SDS.

The time-gated photomultiplier tube (PMT) module H11706-20 and C-mount adapter block were purchased from Hamamatsu Photonics. Optical filters D655/40 and ZET405/20x and dichroic mirror ZT514rc were purchased from Chroma Technology. Laser diode L405P20,

amplifier TIA60, and various optics and mounting parts were purchased from ThorLabs. Non-time-gated fluorescence measurements were performed using a PerkinElmer 2030 microplate reader.

Mn:AgZnInS/ZnS Nanocrystal Based Probes and their Bioconjugation

The synthesis of Mn:AgZnInS/ZnS NCs was performed according to the previously reported methods (Zaeimian et al., 2018). Briefly, Mn-doped AgZnInS NCs were synthesized using 0.2 mmol Zn precursor, 0.2 mmol In precursor, 0.025 mmol Mn precursor, and 0.05 mmol Ag precursor, with a hot injection of 0.8 mmol sulfur precursor at 180 °C. Then, a ZnS shell on NCs was grown to form Mn-doped AgZnInS/ZnS NCs at 210 °C. The Mn:AgZnInS/ZnS NCs were washed out using hexane and ethanol, and dried in vacuum for use.

The produced Mn:AgZnInS/ZnS NCs were transferred to water phase using an amphiphilic polymer (PMAO-CB-SB) as reported in our previous work (Demillo et al.,, 2015). Briefly, we dissolved 30 mg PMAO-CB-SB and 14 mg NCs in an organic solvent, and then added such mixture drop by drop into water under sonication. With sonication, organic droplets containing polymers and NCs were further broken down and dispersed into water. We removed organic solvents using rotary evaporation. During evaporation, self-assembly between PMAO-CB-SB and NCs occurs through hydrophobic interaction while the hydrophilic groups are exposed to aqueous solutions. The water-soluble NC-probes were then filtered using a 0.2 μm filter. Following repeated washed with DI water, the NC-probes were suspended in DI water as a stock (21 mg/mL).

The conjugation protocol used in this study is based on a standard EDC-NHS cross-linking chemistry. First, 25 μL of Mn:AZIS/ZnS stock solution, EDC (20 mg/mL in DI water), and Sulfo-NHS (30 mg/mL in DI water) were combined in a test tube and mixed via vortexing.

Second, 250

μg of monoclonal anti-CPS antibody in PBS was added to the tube, and the mixture was vortexed for 2 hours with intermittent sonication. Third, PBS (1% Tween20) was added into the tube with further vortexing. Next, the solution was isolated via centrifugation and then washed repeatedly with PBS (0.1% Tween20) by resuspending and centrifuging. After removal of the final wash solution, the pelleted conjugate was suspended in 200 μL of PBS-based storage buffer and stored at 4 ˚C for further use.

Signal Synchronization in the Time-Gated Instrument

The signal synchronization in the time-gated instrument was implemented through using a LPC4370 microcontroller unit (MCU) implemented on a LPC-Link2 board. This MCU board has rich programmable resources including a high-quality internal ADC (12-bit, up to 80 MS/s), a dual-core architecture (Cortex M4 with Cortex M0 subsystem), timers, as well as various peripheral circuitries. In the instrument design, there are several time parameters: t_1 corresponds to the length of the excitation pulse applied to the input of the laser driver via GPIO; t_2 defines the length of time after which the PMT gate is turned on, hence the gate delay time is defined by ($t_2 - t_1$); t_3 defines the time at which the ADC begins sampling, thus allowing for a period of ($t_3 - t_2$) for the PMT gate to turn on and for the settling of PMT gate switching noise. Thus, ($t_3 - t_1$) quantifies the sampling delay time. After the desired number of samples have been obtained by the ADC (within the period $t_{sampling}$), the PMT is automatically turned off. The timing for signal synchronization is managed via MCU interrupt handling, which decides among four options depending on the timer value that was reached: (1) when t_1 is reached the excitation pulse signal is ended, thus turning off the laser; (2) when t_2 is reached the PMT gate is on; (3) when t_3 is reached the ADC sampling is started and the desired number of data during a period $t_{sampling}$ are transferred

from the ADC buffer register to a data buffer in memory, after which the PMT gate turns off; (4) when T is reached the timer is automatically reset and the excitation pulse is started if laser pulsing is enabled. In this instrument, the laser diode is pulsed with 200 μs (t_1 = 200 μs) for excitation with a repetition rate of 1.024 kHz, and the sampling delay time after laser-off is 50 μs ($t_3 - t_1$ = 50 μs) and the time period to pick up the signal is 100 μs ($t_{sampling}$ = 100 μs). The detailed instrument design can be referred to our reported work (Gallian et al., 2019).

Conjugation of Microbeads with Monoclonal Antibody

Conjugation of monoclonal anti-CPS antibody to tosylactivated 2.8-μm magnetic beads proceeded according to the previously reported method (Zhu et al., 2010). First, 500 μL of magnetic beads (~15 mg beads) was added into a new microtube. The beads were pull down using magnets, the supernatant (measured to be around 470 μL) was discarded, and 1 mL of Buffer-1 was added into the microtube to wash the beads. After washing (or vortexing) beads in the microtube, the beads were collected using magnets and the supernatant was discarded. Second, 40 μL of stock anti-CPS antibody (~10 mg/mL) and 260 μL of Buffer-1 were added into the microtube to mix with the beads (by vortexing for ~30 seconds or so). 200 μL of Buffer-2 was then added into the microtube to mix with beads. The microtube was then put on a rocker and let the beads and the antibodies incubate at room temperature for 48 hours. Third, after long time incubation, the beads were collected using magnets and the supernatant was discarded. 1 mL of Buffer-3 was added into the microtube to mix with the beads. The beads and Buffer-3 were then incubated for 4~6 hours on the rocker. In this process, BSA in Buffer-3 would react with excess or non-reacted tosyl groups on the bead surfaces and helps block the bead surfaces to minimize the non-specific binding sites. Fourth, after short time incubation, the beads were washed three

times using 1mL of Buffer-4, and resuspended in 470 μL of Buffer-4 to restore the original bead concentration (~ 2 $\times 10^9$ beads/mL). An additional 500 μL of Buffer-4 was added into the microtube to dilute the bead concentration to ~ 1 $\times 10^9$ beads/mL. The conjugated beads were then stored in 4 °C for use.

Immunoassay on CPS

The magnetic bead-based sandwich immunoassay proceeded as follows. Microplates were blocked with 5% milk in PBS prior to use. 2μL of anti-CPS conjugated magnetic beads were added to each well of a microplate, after which the supernatant was removed with the conjugates magnetically fixed to the bottom of the plate. Next, a series of CPS antigen dilutions (diluted in 5% milk in PBS or human serum) were added to the wells and incubated with magnetic beads through vortexing for 60 minutes. After incubation, the beads were washed repeatedly with PBS containing 0.1% Tween20, after which 50 μL of a diluted solution of anti-CPS conjugated NC-probes was added to each well and incubated with vortexing. Next, the NC-Ab conjugate solution was removed, and the beads were again washed repeatedly with PBS containing 0.1% Tween20. 100 μL of an immunoaffinity separation solution was then added to each well and again incubated with vortexing. The supernatant was then transferred to a new microplate for measurement.

Calculation of LOD

The calculation of the immunoassay LOD is based on the following equation:

$$LOD = k \times SD_{blank}/m$$

Where k is a confidence factor, usually 3 for 99% confidence, SD_{blank} is the standard deviation of the blank measurements and m is the slope of the calibration curve (Purushothama et al., 2001).

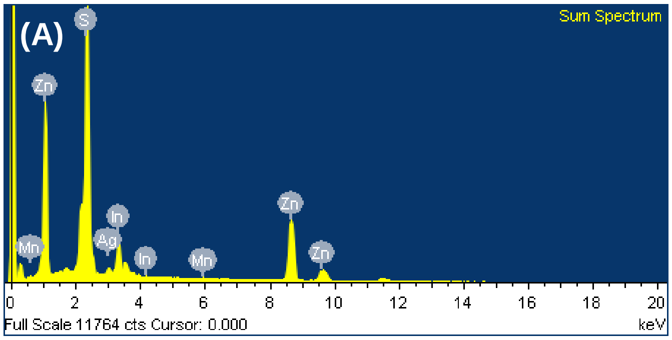

EDX Analysis	Ag	In	Zn	Mn	S
Atomic%	1.13	4.44	44.94	0.41	49.08

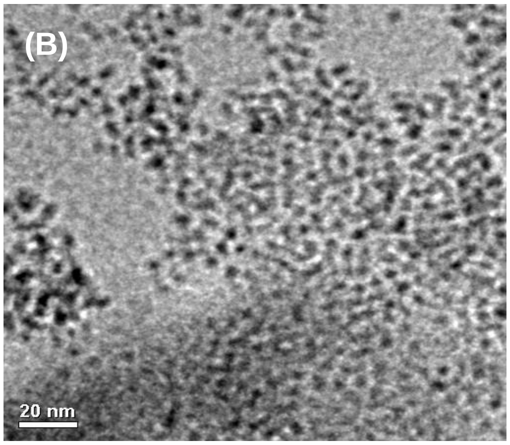

Figure S1. (A) EDX analysis of Mn:AgZnInS/ZnS NCs used in this work. (B) Representative TEM image of Mn:AgZnInS/ZnS NCs.

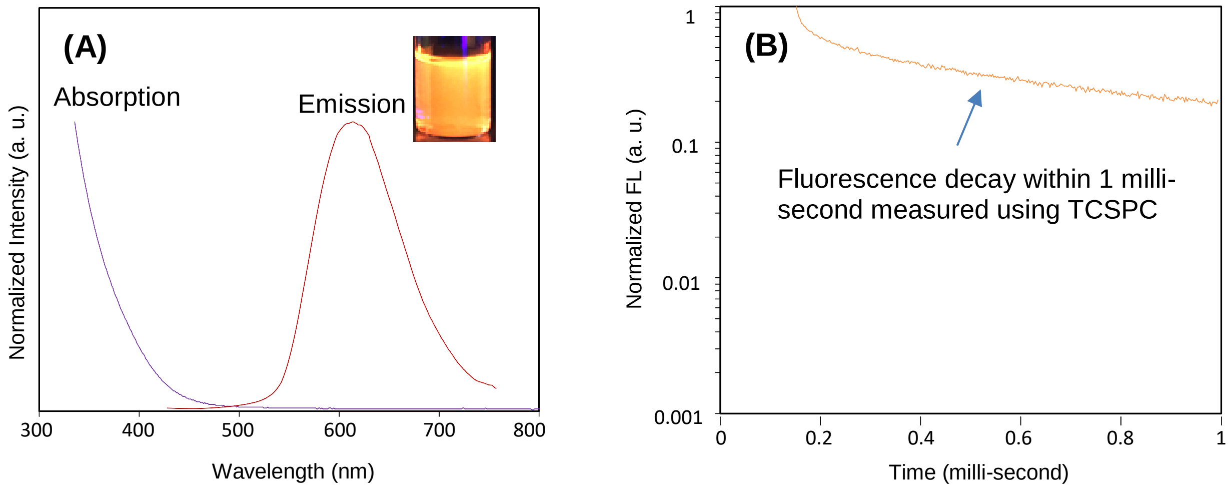

Figure S2. (A) Fluorescence (FL) and absorption spectra of Mn:AgZnInS/ZnS NCs. The inset image presents the NCs suspended in hexane under UV light. (B) Fluorescence decays of Mn:AgZnInS/ZnS NCs measured using time-correlated single photon counting (TCSPC) at the NC emission peak.

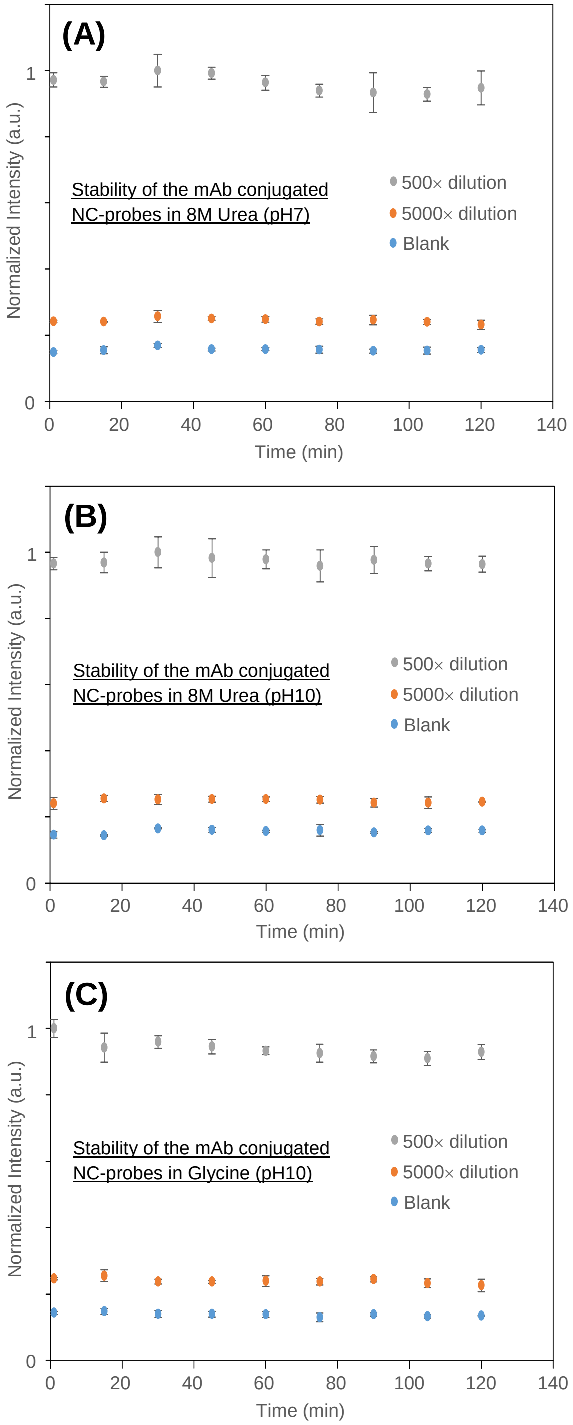

Figure S3. The fluorescence stability of monoclonal anti-CPS conjugated NC-probes with different dilutions from the conjugate stock in different immunoaffinity separation solutions: (A) 8M urea with pH 7, (B) 8M urea with pH10, and (C) 0.1M glycine with pH10. All immunoaffinity separation solutions are supplemented with 0.1% BSA and 1% SDS.

Table S1. Comparison with other reported assays in detecting CPS of *B. Pseudomallei*

Methods	Sample	LOD (pg/mL)	Reference
Gold-nanoparticle based LFIA	Human serum (50 μL)	~ 200	Nualnoi et al., 2016; Houghton et al., 2014
HRP/TMB based ELISA	Buffer (< 300 μL in microplates)	~ 200	Houghton et al., 2014
Gold-nanoparticle based vertical LFIA	Buffer (with a large volume, 50 mL)	~ 20	Chen et al., 2019
Time-Gated Mn:AgZnInS/ZnS NC-probes	Human serum (50 μL in microplates)	~ 23	This work

References:

Chen, P., Gates-Hollingsworth, M., Pandit, S., Park, A., Montgomery, D., AuCoin, D., Gu, J., Zenhausern, F., 2019. Talanta 191, 81–88.

Demillo, V. G., Zhu, X., 2015. J. Mater. Chem. B 3, 8328-8336.

Gallian, B., Dong, G., Zhu, X., 2019. Rev. Sci. Instrum. 90, 104701.

Houghton, R., Reed, D., Hubbard, M., Dillon, M., Chen, H., Currie, B., Mayo, M., Sarovich, D., Theobald, V., Limmathurotsakul, D., Wongsuvan, G., Chantratita, N., Peacock, S., Hoffmaster, A., Duval, B., Brett, P., Burtnick, M., AuCoin, D., 2014. PLOS Negl. Trop. Dis. 8(3): e2727.

Nualnoi, T., Kirosingh, A., Pandit, S. G., Thorkildson, P., Brett, P. J., Burtnick, M., AuCoin, D., 2016. PLOS Negl. Trop. Dis. 10(12): e0005217.

Purushothama, S., Kradtap, S., Wijayawardhana, C., Halsall, H., Heineman, W., 2001. Analyst, 126, 337-341

Zaeimian, M. S., Gallian, B., Harrison, C., Wang, Y., Zhao, J., Zhu, X., 2018. J. Alloys Compd. 765, 236-244.

Zhu, X., Duan, D., Publicover, N., 2010. Analyst 135, 381-389.
